# Time‐course changes in lower limb strength, vertical jump metrics and their relationship with patient reported outcomes following anterior cruciate ligament reconstruction

**DOI:** 10.1002/ksa.12694

**Published:** 2025-05-12

**Authors:** Benjamin Dutaillis, Tyler Collings, Philip Bellinger, Ryan G. Timmins, Morgan D. Williams, Mathew N. Bourne

**Affiliations:** ^1^ School of Health Sciences and Social Work Griffith University Gold Coast Australia; ^2^ Australian Centre for Precision Health and Technology (PRECISE), Menzies Health Institute Queensland Griffith University Gold Coast Australia; ^3^ Griffith Sport Science Griffith University Gold Coast Australia; ^4^ School of Behavioural and Health Sciences Australian Catholic University Brisbane Australia; ^5^ The Sports Performance, Recovery, Injury and New Technologies (SPRINT) Research Centre Brisbane Australia

**Keywords:** ACL, biomechanics, knee reconstruction, rehabilitation

## Abstract

**Purpose:**

To (1) investigate how lower limb strength, vertical jump metrics, and patient reported outcome measures (PROMs) change in the initial 3‐ to 12‐months of recovery following ACLR; and (2) explore which strength and vertical jump metrics best differentiate between lower and higher Knee Osteoarthritis Outcome Score (KOOS) and Anterior Cruciate Ligament Return to Sport After Injury (ACL‐RSI) scores.

**Methods:**

Thirty recreationally active athletes undergoing unilateral primary ACLR underwent field‐based assessments of knee flexion and extension strength, hip adduction and abduction strength, and double and single leg countermovement (CMJ) and drop vertical jump (DVJ) kinetics at 3‐, 6‐, 9‐ and 12‐months following surgery. The KOOS and ACL‐RSI were also completed. Mixed models were used to investigate how measures of lower limb strength, vertical jump metrics and PROMs change across the first 12‐months of rehabilitation. Mixed model decision trees were used to determine which strength and vertical jump measures best discriminated between lower and higher PROMs.

**Results:**

Vertical jump height and reactive strength index (RSI) improved significantly over time (*p* < 0.001), with reductions in contraction and contact times (*p* < 0.05). Isometric knee extension and eccentric knee flexion strength increased significantly (*p* < 0.001), as did KOOS and ACL‐RSI scores (*p* < 0.001). Surgically reconstructed limbs displayed deficits in most vertical jump and strength measures compared to the uninjured contralateral limb, although deficits reduced over time with between limb asymmetry deceasing in 70% of these variables (*p* range: 0.05 to <0.001). Single leg CMJ jump height < 8.4 cm best discriminated between lower and higher ACL‐RSI scores (*r* ^2^ = 0.67, *d* = 0.8), whilst knee extension peak force asymmetry < 38.3% best discriminated between lower and higher KOOS scores (*r* ^2^ = −0.78, *d* = 0.9) across the time‐course of rehabilitation.

**Conclusion:**

Most measures of lower limb strength and vertical jump metrics change in both the ACLR and uninjured contralateral limbs across the first 12‐months of recovery following primary ACLR. A strong relationship was found between measures of lower limb strength and vertical jump performance with PROMs.

**Level of Evidence:**

Level II, lower quality prospective cohort study.

**Abbreviations**
ACLR: anterior cruciate ligament reconstruction
ACL‐RSI: anterior cruciate ligament return to sport index
CMJ: countermovement jump
DVJ: drop vertical jump
KOOS: knee injury osteoarthritis outcome score
PROMS: patient reported outcome measures
RSI: reactive strength index
RTP: return to play
SL: single leg

## INTRODUCTION

Rates of anterior cruciate ligament reconstruction (ACLR) are on the rise worldwide [1, 37]. Following surgery, only half of all individuals return to pre‐injury levels of sports participation [3], and one in five will go on to sustain a second ACL injury [5, 15]. As such, there is a need to improve post‐operative outcomes, particularly for young athletic individuals who wish to safely return to sport.

Field based measures of lower limb strength and vertical jump performance are increasingly popular in clinical practice for ACLR rehabilitation, and recent clinical practice guidelines advocate for their inclusion as part of return to play (RTP) criteria [33]. Vertical jump metrics derived from commercially available portable force‐plate systems are sensitive to deficits in knee joint function at the time‐point of RTP [32, 34]. In addition, field‐based strength testing devices have revealed long‐term deficits in eccentric knee flexor [27] and isometric knee extensor strength [7] that persist long after apparently successful rehabilitation. Despite the call for widespread adoption of these technologies [33], no studies have examined how field based measures of lower limb strength and vertical jump metrics change across the time‐course of rehabilitation, in a single cohort, from early post‐operatively to RTP [18]. Greater insight into how strength and vertical jump metrics in the injured and uninjured contralateral limbs change throughout recovery may enable clinicians to set benchmarks and make more informed decisions during rehabilitation following primary ACLR.

Patient self‐reported outcome measures (PROMs) are key clinical criteria often used as markers of success in patients recovering from ACLR [21, 49]. The Knee injury and Osteoarthritis Outcome Score (KOOS) assesses self‐reported function following knee injuries [47, 50], with lower scores indicating greater pain, difficulty with knee joint function and poor quality of life [47, 50]. The Anterior Cruciate Ligament Return to Sport After Injury (ACL‐RSI) scale was developed to assess an individual's psychological readiness to return to sport following reconstruction [54]. Lower scores are associated with a decreased ability to return to play [43] and increased risk of secondary ACL injury [14]. There is mixed evidence that objective measures of lower limb function are associated with PROMs when assessed at a single time‐point post‐ACLR [2, 4, 13]. However, no studies have explored the relationship between lower limb strength, vertical jump metrics, and KOOS and ACL‐RSI scores across the time‐course of rehabilitation [13, 14, 42, 51, 53, 55]. Identification of objective performance metrics which can discriminate between higher and lower PROMs scores may help to uncover physical capacities underpinning self‐reported function and confidence when returning to sport.

Therefore, the aims of this study were to:

1. Investigate the change in lower limb strength, vertical jump metrics, and PROMs across the first 3‐ to 12‐months of rehabilitation following ACLR.
2. Explore which strength and vertical jump metrics best differentiate between lower and higher KOOS and ACL‐RSI scores across the first 12‐months of recovery following ACLR.

We hypothesised that lower‐limb strength, vertical jump metrics, and PROMS would change non‐uniformly over the time‐course of rehabilitation.

## METHODS

### Participants and study design

An a priori power analysis was conducted using the ‘simr’ package [25] and simulated data to determine the sample size. A general linear model was used in the power analysis rather than a linear mixed effects model due to the unknown random effect parameters. Data were simulated based on a meta‐analysis of unilateral CMJ height after ACLR [18], with an effect size (standardised mean difference) of 0.66 for the effect of limb, and a conservative estimate of 0.22 for the effect time. A minimum sample size of *n* = 20 was estimated to detect a statistically significant limb × time interaction with 80% power. However, a goal sample size of *n* = 30 was set to account for potential participant attrition, and the possibility of a non‐linear effect of ‘time’ across the course of the study period.

Thirty recreationally (*n* = 30) active athletes provided written, informed consent to participate in the study (age = 21.9 ± 5.6, height = 175.7 cm ± 8.0, weight = 74.9 kg ± 12.0), between September 2022 and August 2024. Eligibility criteria required participants to be aged 15–35 years old, have undergone primary unilateral ACLR, and be otherwise free from a history of major lower limb or lower back injuries. Participants were recruited from local allied health and medical clinics in south‐east Queensland, Australia. Participants were engaged in a variety of sports prior to their injury including soccer (*n* = 6), netball (*n* = 5), rugby league (*n* = 4), surfing (*n* = 3), Australian rules football (*n* = 3), rugby union (*n* = 2), cricket (*n* = 2), handball (*n* = 1), strongman (*n* = 1), taekwondo (*n* = 1), oztag (*n* = 1) and basketball (*n* = 1). Most participants underwent ACLR involving a hamstring graft (*n* = 27), while one participant each had a patella, quadriceps, and peroneal tendon graft, respectively. Seven participants had a lateral extra‐articular tenodesis as part of their surgery and 19 participants had concomitant meniscus injuries. All participants underwent reconstruction within three months of sustaining primary ACL rupture.

While rehabilitation was not controlled by the research team, all participants undertook 6‐12 months of a criteria‐based rehabilitation program under the guidance of a qualified physiotherapist. Participants reported engaging in resistance and plyometric exercise aimed at restoring lower limb strength, muscle mass and motor control, as well as a graduated return to sport specific training. Participants were invited to complete testing at the following four regular time‐points across the first year after surgery: 3‐ (range 3–4), 6‐ (range 6–7), 9‐ (range 9–10) and 12‐months (range 12–13). This study was approved by the University Human Research Ethics Committee (Ref No: 2022/253).

### Data collection

At the start of every testing session all participants completed both the KOOS and ACL‐RSI questionnaires. Following this, participants completed a standardised warm‐up consisting of 5 mins of stationary cycling at a self‐selected low‐moderate intensity, followed by 10 body weight squats and 10 CMJs. Vertical jump testing was completed on a ForceDecks (VALD, Brisbane) dual portable force‐plate system sampling at 1000 Hz. Vertical jump testing occurred in the following order from low to high intensity: double leg CMJ, single leg CMJ, double leg DVJ, single leg DVJ, as per previous studies [34]. However, due to safety concerns, both double and single leg DVJs were only completed from the 6‐month time‐point onwards. Participants were given a demonstration and 2–3 practices of each jump type prior to recording of trials to familiarise them. The force‐plates were then zeroed, and the participant's body weight was recorded, as per manufacturers procedures. Misidentified or poor‐quality jumps (i.e., landing outside the bounds of the force‐plates, use of arms or loss of balance on landing) were manually removed by the tester. Participants performed three trials of each jump type with a minimum of 30 s rest between jumps and jump types.

Following vertical jump testing, participants underwent assessments of isometric knee extension, eccentric knee flexion and, isometric hip abduction and adduction strength using the Dynamo, Nordbord and ForceFrame (VALD, Brisbane, Australia), respectfully. The order of strength testing (and limbs for the isometric knee extension test) were randomised to ensure no bias of fatigue. Participants were given a demonstration and 2–3 50% effort practices of each test type to familiarise them. Participants performed three trials of each strength test with a minimum of 30 s rest between trials and test types. The testing battery was co‐designed with allied health practitioners from south‐east Queensland who specialise in ACL injuries. All testing was completed under the supervision of a qualified allied health professional (BD).

#### Double and single leg CMJ

Countermovement jumps began with participants standing tall and still with their hands on their hips. Participants were given a three second countdown prior to jumping and instructed to jump as high and fast as possible, using a self‐selected depth, while keeping their legs as straight as possible in the air. For double leg CMJs, participants had to take‐off and land with one foot on each force‐plate. For single leg CMJs, participants had to take‐off and land on a single force‐plate with the testing limb whilst the free limb remained off the ground [34]. Based on the findings from recent literature [12] and current RTP criteria [33], eccentric and concentric peak force and impulse of each limb during the double leg CMJ, along with jump height, contraction time and RSI for both single and double leg jumps were selected for subsequent analysis. These metrics have demonstrated good to excellent reliability (intraclass correlation coefficient (ICC) = 0.85–0.96) [8, 12, 44].

#### Double and single leg DVJ

Participants were asked to stand tall with their hands on their hips and roll and drop from a 30 cm box. Upon hitting the ground, they were instructed to immediately jump as high and fast as possible, minimising time spent on the ground, while keeping their knees extended in the air. For double leg DVJs, participants rolled and dropped off the box leading with a self‐selected leg and had to land and take‐off with one foot on each force‐plate. For single leg DVJs, participants rolled and dropped off the box leading with their testing limb and had to land and take‐off from a single force‐plate whilst the free limb remained bent (i.e., knee flexed) to avoid touching the ground [34]. Based on the findings from recent literature [12] and current RTP criteria [33], eccentric and concentric peak force and impulse of each limb during the double leg DVJ, along with jump height, contact time and RSI for both single and double leg DVJs were selected for subsequent analysis. These metrics have demonstrated good to excellent reliability (intraclass correlation coefficient (ICC) = 0.87–0.97) [8, 12].

#### Knee extension strength

Isometric knee extension strength was measured using a handheld dynamometer set up in tension format (Dynamo, VALD, Brisbane, Australia), independently for both legs. Participants were seated on a bench with their hips and knees flexed at 90° [7]. The Dynamo was secured to their ankle superior to the lateral malleolus using the manufacturers supplied ankle strap, and horizontally behind them via rigid straps (sampling at 225 Hz). Participants were instructed to securely hold themselves down to the bench they were seated on and ‘kick’ as hard as possible for 5‐s to achieve a maximum voluntary isometric contraction (MVIC). Strong verbal encouragement was given during all testing. Knee extension testing procedures using handheld dynamometers have previously shown excellent reliability (ICC = 0.93–0.98) [26, 30].

#### Eccentric knee flexion strength

Eccentric knee flexion strength was assessed during the Nordic hamstring exercise (NordBord, VALD, Brisbane, Australia). Participants were positioned in a kneeling position on the NordBord, with their ankles secured immediately superior to the lateral malleoli by individual ankle hooks attached to uniaxial load cells (sampling at 50 Hz). Participants were instructed to gradually lean forward at a slow speed, aiming to get ‘long and low’, while maximally resisting this movement with both lower limbs, and keeping the trunk and hips in a neutral position throughout with hands held across the chest. Strong verbal encouragement was given during all testing. Eccentric knee flexion strength was measured from the left and right legs independently. These testing procedures have shown good to excellent reliability (ICC = 0.83–0.90) [43].

#### Hip adduction and abduction strength

Isometric hip abduction and adduction strength were assessed independently for the left and right legs using dual frame‐fixed dynamometers (ForceFrame, VALD, Brisbane, Australia) (sampling rate: 50 Hz). Participants lay supine, with their hips in neutral and knees fully extended, and their medial and lateral malleoli aligned to the load cells [11]. They were then instructed to ‘squeeze’ (i.e., adduct their hip) and ‘push’ (i.e., abduct their hip) as hard as possible for 5‐s to achieve an MVIC. Strong verbal encouragement was given during all testing. Participants performed three maximal voluntary isometric contractions, alternating between hip abduction and adduction with 5‐ to 10‐s rest in between efforts. Previous work employing a similar testing protocol has shown excellent reliability (ICC = 0.94) [47].

#### Patient reported outcome measures

The KOOS is a 42 item questionnaire that evaluates knee specific function and quality of life in persons with any knee injury, across five subscales: pain, function, activities of daily living, sport and recreation, and quality of life [45]. KOOS score and subscales scores have demonstrated moderate to excellent reliability (ICC = 0.75–0.93) [48]. The ACL‐RSI is a 12 item questionnaire designed to measure an individual's psychological readiness to return to sport following ACLR [52]. It has shown excellent reliability for use in individuals following ACLR (Cronbach's alpha = 0.92) [52].

### Data analysis

Total scores from the KOOS and ACL‐RSI, along with selected metrics for each individual trial from the strength and vertical jump tests were exported from ForceDecks, Dynamo, Nordbord and ForceFrame software, then imported into Rstudio. For all vertical jump metrics, the mean of three trials was used for analyses [11, 34, 46]. For all strength tests, the peak force of the three trials in each limb was identified and used for all subsequent analysis [11]. Between limb asymmetry was calculated using the following formulas [9]:

Double leg jumps:

(uninjured limb–ACLR limb)/(uninjured limb+ACLR limb)×100.

Single leg jumps and lower limb strength measures:

(uninjured limb–ACLR limb)/uninjured limb×100.

Positive values indicate that the uninjured limb was greater than the ACLR limb.

### Statistical analysis

All statistical analyses were conducted in R Studio (R version 4.2.0). Data were reported descriptively as means ± standard deviation (SD), and mean change scores between timepoints with 95% confidence intervals (CI). To examine the time‐course changes in KOOS, ACL‐RSI, lower limb strength and vertical jump metrics across the first year of recovery, a linear mixed effects model with time by limb as an interaction fixed effect and participant as a random effect were fitted for each metric using the ‘lme4’ package [16]. For models examining KOOS, ACL‐RSI, and double leg CMJ and DVJ jump height, contact/contraction time and RSI, limb was removed as a predictor. A second model was then fitted with the same fixed (i.e., time by limb) and random (i.e., participant) effects, with a natural cubic spline applied to the time variable to model any non‐linear change over the time‐course of rehabilitation [20]. These two models were then compared via ANOVA using the ‘anova’ function and the strongest model (based on an identified significant difference *p* < 0.05, and < 5 BIC) was kept for reporting. Effect sizes for the change from baseline to 12‐months post‐operatively (separately for each limb where appropriate) were calculated from the models and interpreted as small (*d* = 0.2), medium (*d* = 0.5) and large (*d* ≥ 0.8) [10]. As this was a within person, between limb analysis, no metrics were normalised to body weight as this does not influence the model outcomes.

Decision tree induction was used to determine which strength and vertical jump measures best discriminated between lower and higher PROMs. Mixed model decision trees offer a flexible tool for exploring the relationship between continuous variables in health research, while accounting for the repeated measures data structure of longitudinal studies [22]. Separate decision trees with a global fixed effect of ‘time’ and a random effect of ‘participant’ were built using the ‘glmertree’ package [22] for KOOS and ACL‐RSI scores. ACLR limb and between limb asymmetry values for all lower limb strength, CMJ and single leg CMJ metrics were input as potential partitioning variables (local node fixed effects). DVJ force‐time metrics were removed from this analysis given they were not performed at the 3‐month timepoint. The alpha value was set at 0.05 with a Bonferroni correction applied. Effect sizes between groups in terminal nodes were then calculated [54] and interpreted as small (*d* = 0.2), medium (*d* = 0.5) and large (*d* ≥ 0.8) [10].

## RESULTS

Of the 30 participants involved in the study, 19 (*n* = 7 female) completed testing at all four time‐points. However, 23 (*n* = 8 female), 27 (*n* = 10 female), 25 (*n* = 9 female) and 23 (*n* = 9 female) participants took part in the 3‐, 6‐, 9‐, and 12‐month post‐operative testing sessions, respectively. Of the 23 participants who completed the 12‐month test, 19 had returned to playing their respective sport.

### Mixed model analysis

**Figure 1:**
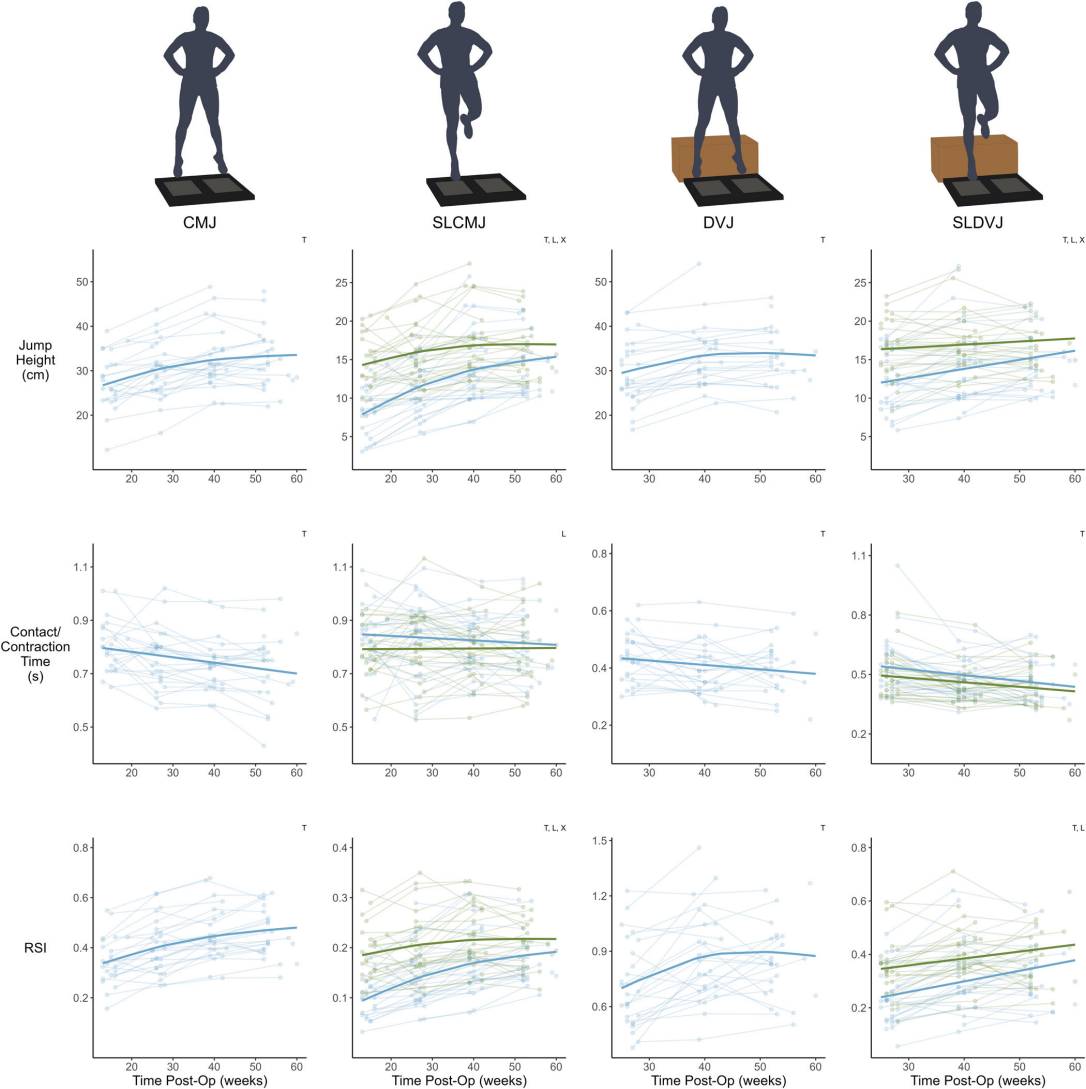
Time‐course changes in jump height, contact/contraction time and reactive strength index (RSI) for countermovement jump (CMJ), single leg CMJ (SLCMJ), drop vertical jump (DVJ) and single leg DVJ (SLDVJ). For SLCMJ and SLDVJ, green = uninjured limb, blue = ACLR limb. Lighter points and adjoining lines represent individual data points and darker lines show the mixed model time‐course trajectory. Significant effects (*p* < 0.05) of the mixed models are indicated in top right of each plot (T = time, L = limb, and X = time × limb). ACLR, anterior cruciate ligament reconstruction.

**Figure 2:**
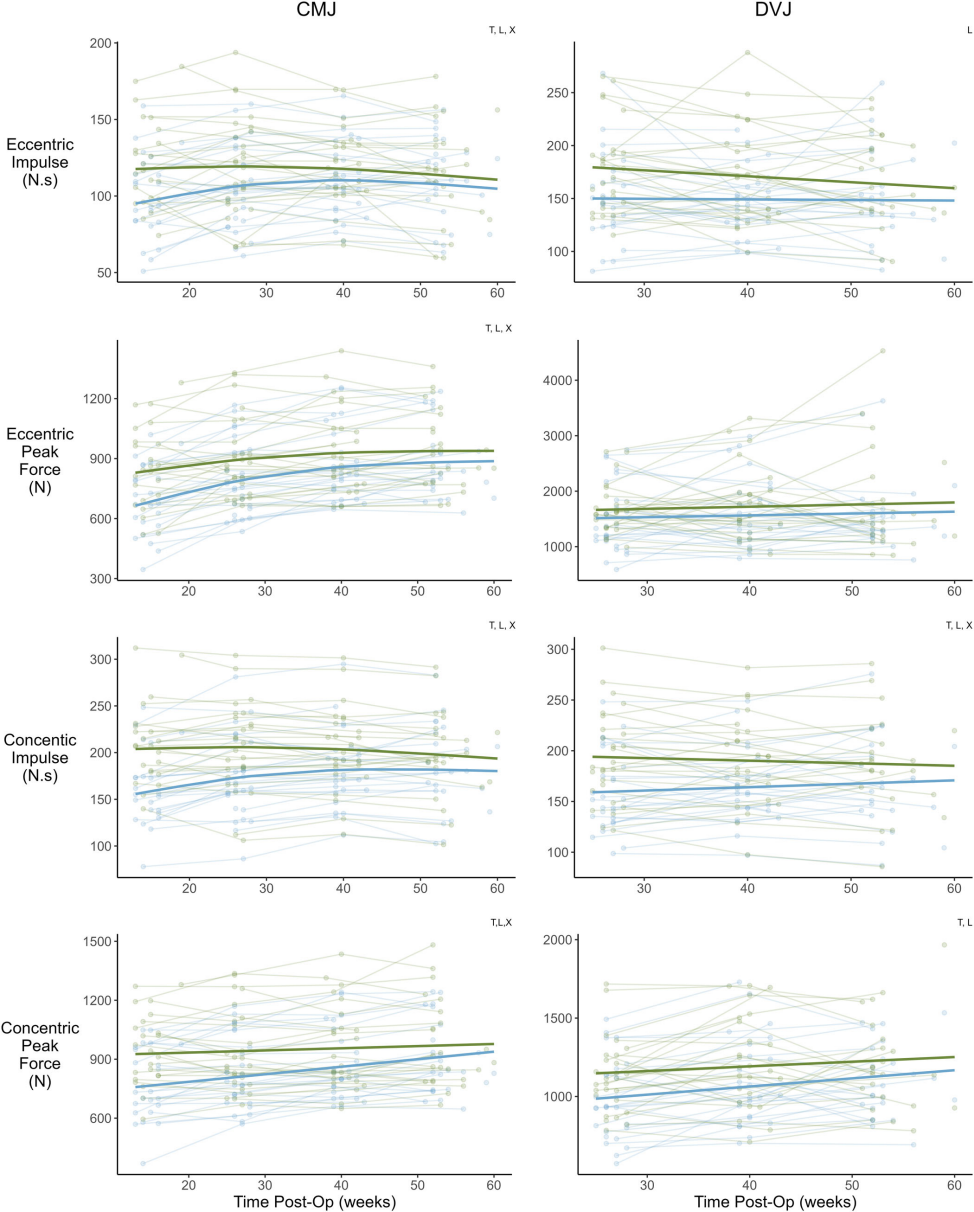
Time‐course changes in countermovement jump (CMJ) and drop vertical jump (DVJ) force metrics. Green = uninjured limb, blue = ACLR limb. Lighter points and adjoining lines represent individual data points and darker lines show the mixed model time‐course trajectory. Significant effects (*p* < 0.05) of the mixed models are indicated in top right of each plot (T = time, L = limb, and X = time × limb). ACLR, anterior cruciate ligament reconstruction.

**Figure 3:**
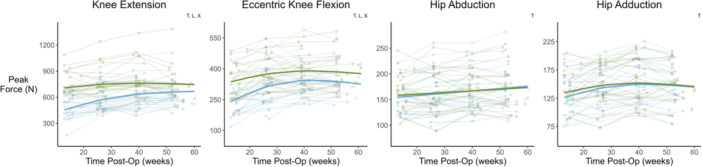
Time‐course changes in lower limb strength measures. Green = uninjured limb, blue = ACLR limb. Lighter points and adjoining lines represent individual data points, whilst the darker lines show the mixed model time‐course trajectory. Significant effects (*p* < 0.05) of the mixed models are indicated in top right of each plot (time = T, limb = L, and time × limb = X). ACLR, anterior cruciate ligament reconstruction.

**Figure 4:**
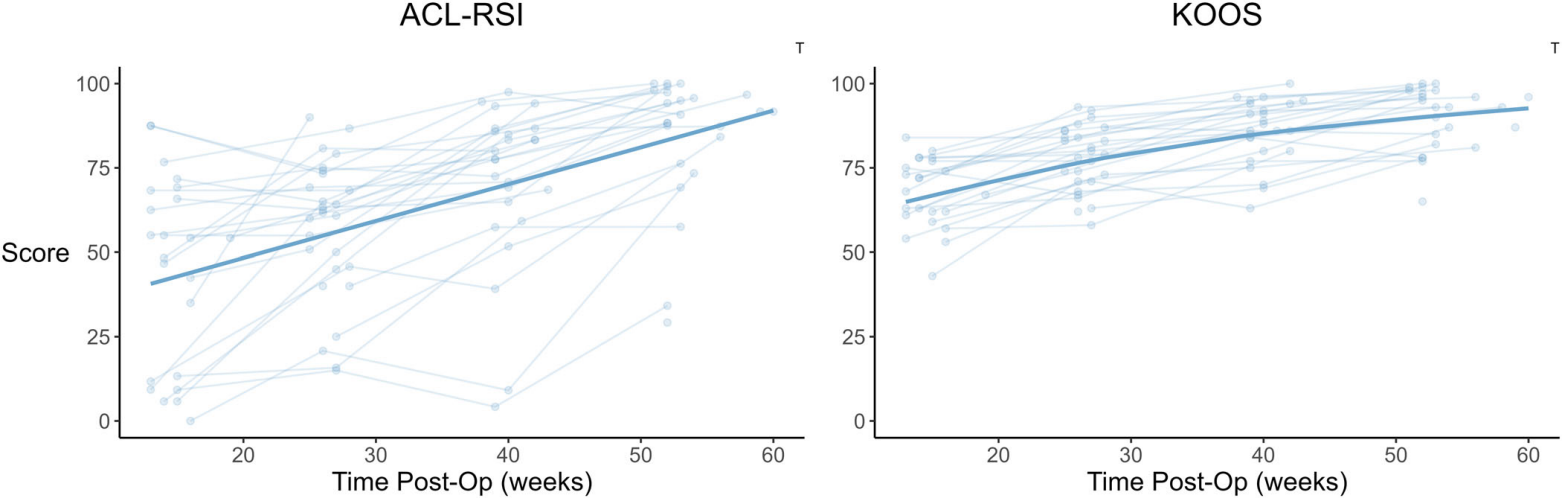
Time‐course changes in anterior cruciate ligament return to sport after injury (ACL‐RSI) score and Knee Injury and Osteoarthritis Outcome Score (KOOS). Lighter points and adjoining lines represent individual data points, whilst the darker lines show the mixed model time‐course trajectory. Significant effects (*p* < 0.05) of the mixed models are indicated in top right of each plot (T = time).

The change in CMJ jump height, RSI, concentric impulse, eccentric impulse and eccentric peak force, DVJ height and RSI, SLCMJ height and RSI, isometric knee extension, eccentric knee flexion and isometric hip adduction strength, and KOOS followed a non‐linear trajectory over time. The remaining variables were best fitted with linear models (Figures 1, 2, 3, 4, Table 1). For all metrics, baseline data (mean ± SD), change scores between consecutive testing time‐points (mean and 95% CI), and effect size (Cohen's *d*) for the change from baseline to 12‐months post‐operatively are reported in Table 1.

**Table 1:** Changes in all vertical jump metrics, strength and patient‐reported outcome measures across the time‐course of recovery following primary anterior cruciate ligament reconstruction.

	Baseline data^a^		3–6 months		6–9 months		9–12 months		Baseline to 12‐months	
	Mean ± 1 SD		Mean [95%CI]		Mean [95%CI]		Mean [95%CI]		Effect size (*d*) [95%CI]	
Double leg jump performance										
CMJ jump height (cm)^b^	27.5 ± 6.3		3.0 [1.8–4.2]		2.8 [1.6–4.0]		0.5 [−0.7, 1.7]			2.9 [2.0–3.8]
CMJ contraction time (s)^c^	0.80 ± 0.10		−0.03 [−0.07, 0.01]		−0.02 [−0.04, 0.00]		−0.02 [−0.05, 0.01]			−1.7 [−0.9, −2.5]
CMJ RSI^b^	0.35 ± 0.10		0.06 [0.03–0.09]		0.05 [0.03–0.07]		0.02 [0.00–0.04]			3.7 [2.8–4.6]
DVJ jump height (cm)^b^	29.7 ± 7.0					3.6 [2.1–5.1]		0.4 [−0.5, 1.3]		1.8 [0.8–2.7]
DVJ contact time (s)^c^	0.44 ± 0.08					−0.04 [−0.08, 0.00]		−0.01 [−0.05, 0.03]		−0.9 [−0.1, −1.7]
DVJ RSI^b^	0.71 ± 0.23					0.16 [0.09–0.23]		0.02 [−0.06, 0.10]		1.4 [0.5–2.3]

	ACLR	Uninjured	ACLR	Uninjured	ACLR	Uninjured	ACLR	Uninjured	ACLR	Uninjured
Single leg jump performance										
SLCMJ jump height (cm)^b^	8.7 ± 3.6	14.9 ± 3.7	2.7 [1.9–3.5]	0.9 [−0.2, 2.1]	2.9 [1.8–3.9]	1.6 [0.7–2.5]	1.1 [0.4–1.9]	−0.3 [−0.9, 0.3]	4.1 [3.2–4.8]	1.4 [0.7–2.2]
SLCMJ contraction time (s)	0.84 ± 0.13	0.79 ± 0.10	0.01 [−0.04, 0.06]	0.03 [−0.01, 0.07]	−0.02 [−0.06, 0.02]	−0.02 [−0.05, 0.01]	−0.02 [−0.06, 0.02]	0.01 [−0.02, 0.04]	−0.6 [−1.2, 0.1]	−0.1 [−0.6, 0.7]
SLCMJ RSI^b^	0.10 ± 0.04	0.19 ± 0.05	0.03 [0.02–0.04]	0.01 [−0.01, 0.03]	0.04 [0.02–0.06]	0.02 [0, 0.04]	0.02 [0.01–0.03]	−0.01 [−0.02, 0]	3.4 [2.6–4.2]	1.1 [0.4–1.8]
SLDVJ jump height (cm)^c^	12.2 ± 4.0	16.4 ± 3.8			2.4 [1.2–3.7]	1.1 [0.1–2.1]	1.2 [0.3–2.1]	−0.3 [−1.1, 0.5]	2.4 [1.5–3.2]	0.80 [0.1–1.6]
SLDVJ contact time (s)^c^	0.55 ± 0.14	0.49 ± 0.11			−0.07 [−0.12, −0.02]	−0.05 [−0.08, −0.02]	−0.01 [−0.05, 0.03]	0.00 [−0.04, 0.04]	−1.5 [−0.8, −2.3]	−1.2 [−0.4, −2.0]
SLDVJ RSI^c^	0.24 ± 0.11	0.35 ± 0.11			0.08 [0.05– 0.11]	0.06 [0.03– 0.09]	0.04 [0.01– 0.07]	0.00 [−0.04, 0.04]	2.4 [1.5–3.2]	1.54 [0.8–2.3]
Double leg jump force metrics										
CMJ concentric impulse (N.s)^b^	162 ± 41	210 ± 43	18 [11–25]	1 [−6, 7]	7 [2–12]	−2 [−6, 1]	2 [−4, 9]	−7 [−12, −2]	1.8 [1.0–2.5]	−0.74 [0.0, −1.5]
CMJ concentric peak force (N)^b^	774 ± 183	954 ± 185	66 [31– 102]	−12 [−42, 17]	46 [22–69]	14 [−13, 41]	44 [15–73]	19 [−15, 54]	2.6 [1.8–3.3]	0.7 [0.0–1.4]
CMJ eccentric impulse (N.s)^b^	100 ± 28	122 ± 29	12 [6–17]	1 [−7, 8]	3 [−1, 7]	−2 [−8, 4]	−2 [−7, 3]	−5 [−12, 2]	0.8 [0.1–1.6]	−0.6 [−1.3, 0.1]
CMJ eccentric peak force (N)^b^	697 ± 179	858 ± 206	120 [86–155]	50 [3–96]	62 [37–88]	22 [−13, 57]	35 [3–66]	13 [−18, 45]	2.8 [2.0–3.5]	1.4 [0.6–2.1]
DVJ concentric impulse (N.s)^c^	159 ± 39	193 ± 45			5 [−5, 14]	−5 [−12, 3]	6 [−4, 16]	−4 [−12, 5]	0.8 [0.0–1.5]	−0.6 [−1.3, 0.2]
DVJ concentric peak force (N)^c^	972 ± 251	1128 ± 233			111 [50–171]	81 [−1, 163]	44 [−37, 125]	−11 [−100, 78]	1.3 [0.6–2.1]	0.8 [0.0–1.5]
DVJ eccentric impulse (N.s)	151 ± 41	177 ± 42			−6 [−18, 7]	−9 [−23, 5]	6 [−7, 20]	−10 [−25, 4]	−0.1 [−0.7, 0.8]	−0.7 [−1.4, 0.0]
DVJ eccentric peak force (N)	1515 ± 612	1661 ± 444			77 [−120, 275]	79 [−142, 301]	48 [−154, 250]	6 [−277, 290]	0.3 [−0.4, 1.0]	0.3 [−0.4, 1.1]
Lower limb strength										
Knee extension (N)^b^	491 ± 134	738 ± 156	93 [56–129]	10 [−26, 46]	80 [53–106]	47 [17–77]	26 [−13, 65]	−42 [−85, 0]	2.7 [1.9–3.5]	0.50 [−0.2, 1.2]
Eccentric knee flexion (N)^b^	255 ± 91	345 ± 85	66 [45–87]	36 [14–58]	25 [1–49]	2 [−21, 25]	−1 [−18, 16]	10 [−6, 26]	2.1 [1.3–2.8]	1.0 [0.3–1.7]
Hip abduction (N)^c^	156 ± 34	163 ± 36	10 [2–19]	1 [−8, 10]	5 [−1, 12]	9 [0–18]	3 [−4, 9]	0 [−9, 8]	1.7 [0.9–2.4]	1.2 [0.4–1.9]
Hip adduction (N)^b^	134 ± 38	142 ± 31	9 [−2, 20]	8 [−1, 18]	14 [7–21]	9 [0–17]	−6 [−16, 4]	−4 [−14, 5]	1.2 [0.5–2.0]	0.7 [0.0–1.5]
PROMs										
KOOS total^b^	68 ± 10		11 [7–15]		8 [5–11]		7 [4–9]		5.0 [4.0–6.0]	
ACL‐RSI^c^	45 ± 28		15 [6–24]		15 [8–21]		17 [10–25]		3.5 [2.7–4.4]	

Abbreviations: ACLR, anterior cruciate ligament reconstruction; CI, confidence interval; SD, standard deviation.

^a^ Single and double‐leg countermovement jump (CMJ) baseline at 3 months. Single and double‐leg drop vertical jump (DVJ) baseline at 6 months.

^b^ Non‐linear change over time.

^c^ Linear change over time.

#### Time‐course trajectories for vertical jump metrics, strength and PROMs

For all jump types, both jump height and RSI increased over time (*p* < 0.001). In contrast, contraction time for CMJ, and contact time for DVJ and SLDVJ, reduced over time (*p* < 0.05). All CMJ double leg force metrics increased with time (*p* < 0.001), along with DVJ concentric impulse and concentric peak force (*p* < 0.05). See Figures 1 and 2, and Table 1.

All strength variables increased over time (*p* < 0.001). Both KOOS and ACL‐RSI scores increased over time (*p* < 0.001). See Figure 3 and Table 1.

#### Between limb differences in vertical jump metrics and strength

Compared to the contralateral uninjured limb, the ACLR limb displayed deficits in all vertical jump metrics (*p* range: 0.014 to <0.001), except double leg DVJ eccentric peak force and single leg DVJ contact time (Figures 1 and 2, Table 1). Additionally, deficits in eccentric knee flexion and isometric knee extension strength (*p* < 0.001) were observed in the ACLR limb (Figure 3, Table 1).

#### Time by limb interaction

**Figure 5:**
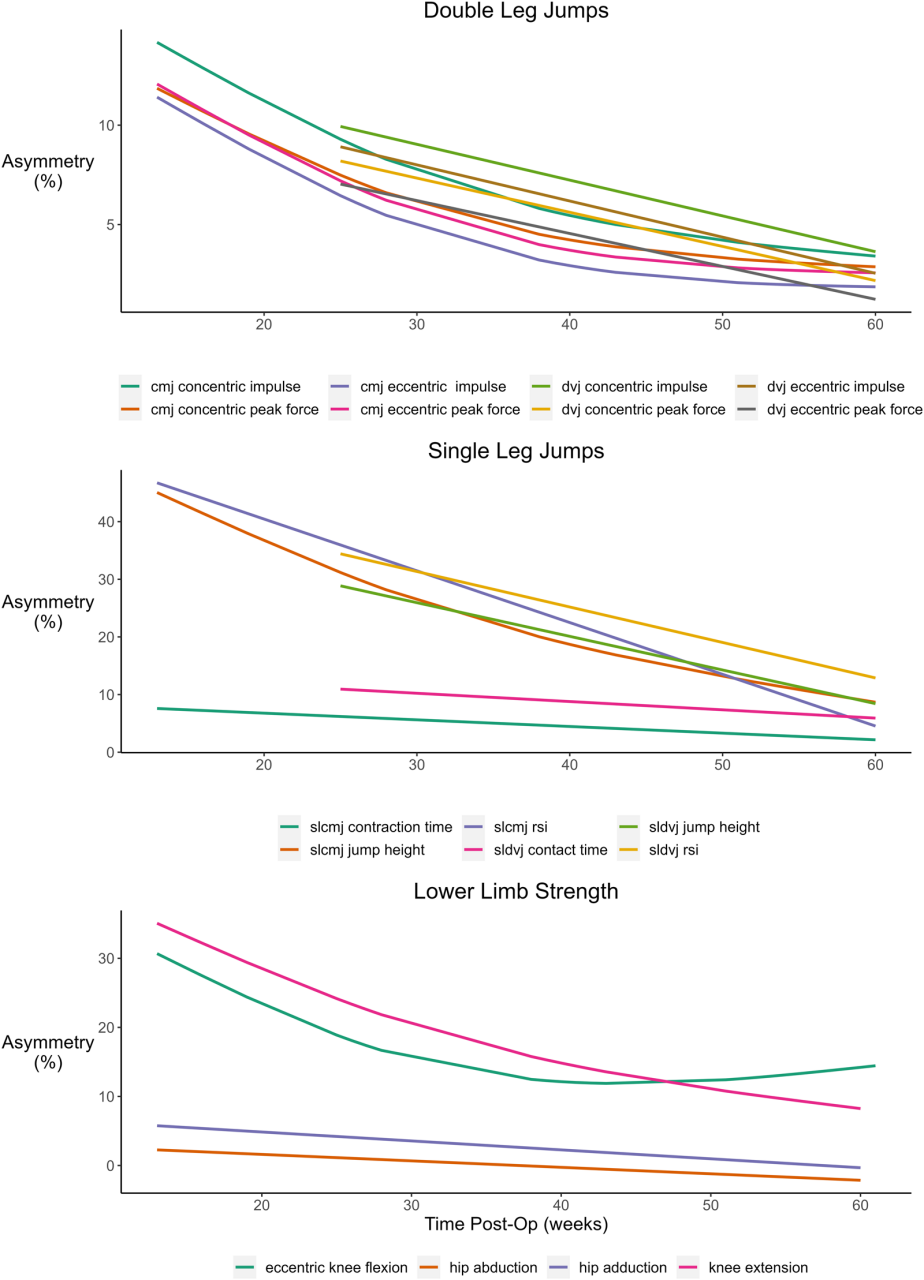
Time‐course changes in between limb asymmetry for double leg (top) and single leg (middle) countermovement jump (CMJ) and drop vertical jump (DVJ) metrics, along with lower limb strength (bottom) measures.

Between limb asymmetry reduced over time, as indicated by the significant time by limb interaction (*p* < 0.05), for all double leg CMJ metrics (*p* range: 0.075 to <0.001), single leg CMJ jump height (*p* < 0.001) and RSI (*p* < 0.001), double leg DVJ concentric impulse (*p* < 0.001), single leg DVJ height (*p* < 0.001), as well as isometric knee extension (*p* < 0.001) and eccentric knee flexion strength (*p* < 0.001). See Figures 1, 2, 3, 4, 5, and Table 1.

### Mixed model decision trees

**Figure 6:**
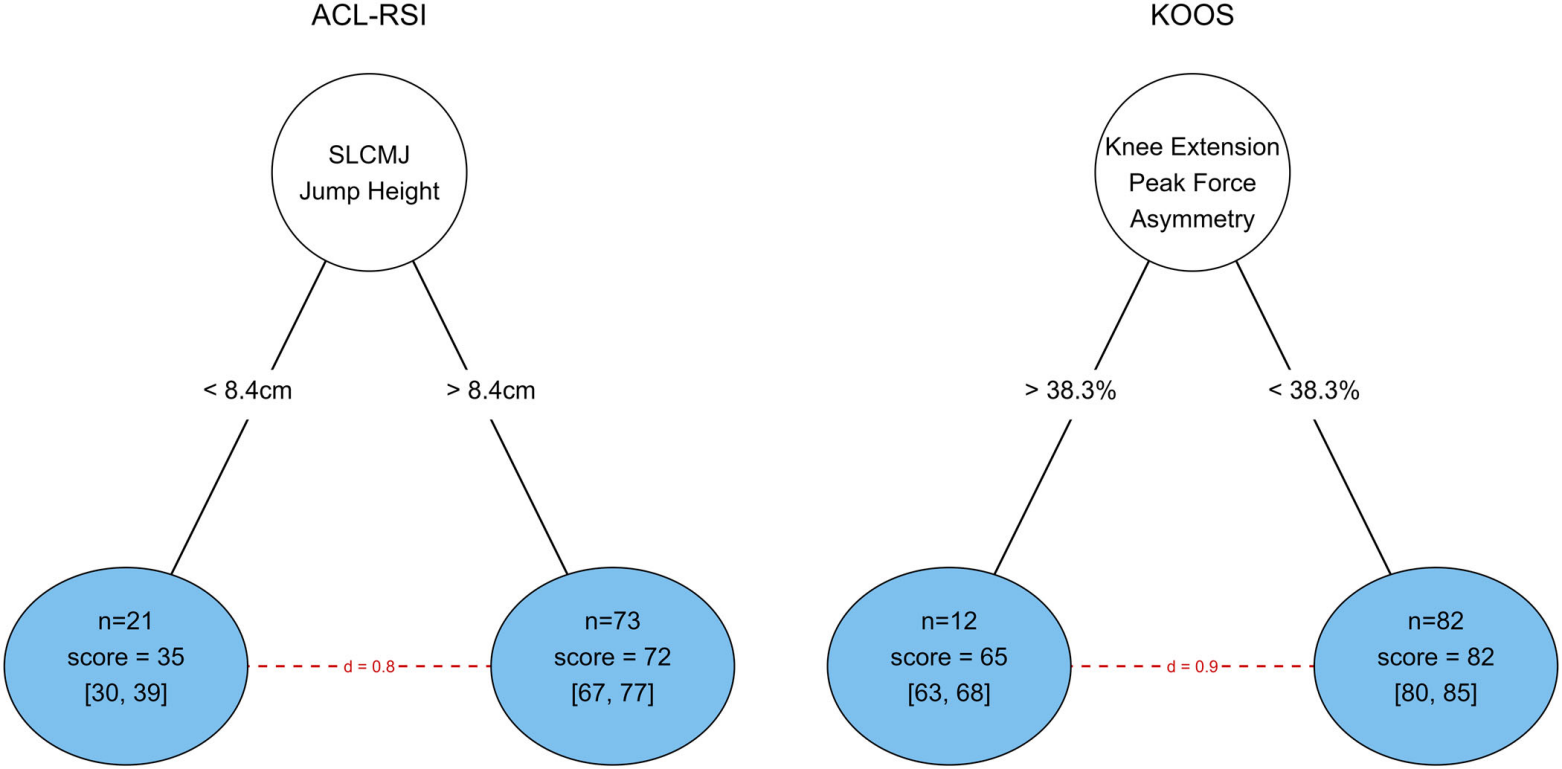
Mixed model decision trees for anterior cruciate ligament return to sport after injury (ACL‐RSI) score and Knee injury and Osteoarthritis Outcome Score (KOOS). Terminal nodes (blue) show the number of observations and mean score [95% confidence interval]. Red numbers indicate the between node effect size (Cohen's *d*). SLCMJ, single leg countermovement jump.

For ACL‐RSI score, the decision tree model returned a single partitioning variable: single leg CMJ jump height for the ACLR limb that explained 67% of variance in the ACL‐RSI score (*r* ^2^ = 0.67), with a cut‐off value of 8.4 cm (Figure 6). There was a large effect size (*d* = 0.8) between nodes. For KOOS score, a single partitioning variable was selected: isometric knee extension peak force asymmetry, that explained 78% of variance in KOOS score (*r* ^2^ = 0.78), with a cut‐off value of 38.3%. A large effect size (*d* = 0.9) was observed between nodes (Figure 6).

## DISCUSSION

The results largely supported our initial hypothesis. By 12‐months post‐ACLR, most vertical jump metrics for the reconstructed limb approached values observed in the uninjured contralateral limb. Isometric knee extension and eccentric knee flexion strength of both limbs increased, whereas isometric hip abduction and adduction strength were not different between limbs, and changed very little over time. Both ACL‐RSI and KOOS scores also increased from 3‐ to 12‐months. Among the performance metrics, single leg CMJ jump height best discriminated between lower and higher ACL‐RSI scores, whilst knee extension peak force asymmetry best differentiated higher and lower KOOS score across the time‐course of rehabilitation. This is the first study to longitudinally assess changes in vertical jump metrics, lower limb strength and PROMs, concurrently, from 3‐ to 12‐months following primary ACLR. These data provide unique clinical insight into the recovery trajectories of objective and subjective measures following ACLR.

Our findings show a general trend towards faster recovery in double leg jump force metrics, jump height and RSI in the ACLR limb during the first approximately 6‐months of rehabilitation, then a continued slower recovery up to 12‐months post‐operatively. Similarly, isometric knee extension and eccentric knee flexion peak force improved more rapidly in early compared to late rehabilitation (Figure 3, Table 1). These findings align with a recent meta‐analysis which suggested more rapid recovery of isokinetic knee flexor and extensor strength during early compared to late rehabilitation [23]. However, we are the first to report equivalent trajectories in measures of vertical jump performance. The mechanisms underpinning these recovery trajectories are not fully understood, but might be explained by a number of maladaptations in structure and function following ACLR. Deficits in quadriceps and hamstring muscle size [19] and voluntary activation [50] are well documented in early rehabilitation from ACLR and contribute to reduced muscle force producing capacity [17, 24, 31, 35, 40]. The early recovery trends observed here presumably reflect diminishing returns as patients overcome such deficits throughout rehabilitation. These findings provide valuable benchmarks, allowing clinicians to identify athletes diverging from common recovery trajectories, and intervene early with targeted rehabilitation strategies.

While the vertical jump force and jump height metrics across all jumps generally improved over the time‐course of rehabilitation, contact and contraction time displayed very little change. Double leg CMJ contraction time and double and single leg DVJ contact time showed less then approximately 0.1 s decrease across the first 12 months of recovery, while single leg CMJ contraction time displayed no change at all. Additionally, there was little to no between limb differences in contraction/contact time during both single leg CMJ and DVJ across the time‐course of rehabilitation. These data support the findings of a recent systematic review and meta‐regression [18] which revealed that single leg DVJ contact time did not differ between limbs with and without a history of ACLR, nor change over time after injury. However, our results also revealed an increase in RSI across all jump types. Given that RSI is the quotient of jump height and contact/contraction time [29, 34, 46], it appears that improvements in RSI are largely modulated by increased jump height. Current RTP criteria suggest RSI values of >0.5 and >1.3 for double and single leg DVJ, respectfully. However, it is unclear whether monitoring RSI provides additional information to clinicians, as opposed to jump height alone.

Our findings suggest that between limb asymmetry in force metrics from double leg CMJ and DVJs decrease similarly over rehabilitation (Figure 5, Table 1), with all metrics having on average, <10% asymmetry at the 12‐month timepoint. These findings are in contrast to prior work which suggested asymmetrical jumping strategies are most pronounced during the concentric phase [34], and that there may be a difference in the recovery of between limb symmetry depending on the jump type and metric chosen [18]. A recent systematic review and meta‐regression demonstrated that CMJ eccentric and concentric peak forces recover over time following ACLR, but CMJ eccentric and concentric impulse and DVJ eccentric and concentric peak forces do not [18]. However, this is the first study to track a single cohort of patients at four regular time intervals across the first year following primary ACLR, which may explain the difference in findings. Recent clinical practice guidelines recommend using double leg CMJs and DVJs to assess asymmetrical jump strategies and suggest that <10% between limb asymmetry for eccentric and concentric impulse is used as RTP criteria [33]. Our results suggest that no further information is gained by looking across jump types and metrics. Further work is needed to clarify if current RTP testing protocols can be refined to reduce the time burden on practitioners and patients.

The results of this study suggest that strength and jump performance in the uninjured contralateral limb can change considerably in the first year of recovery follow ACLR. These data support recent observations of increased knee extension and flexion strength in the uninjured limb across the time‐course of rehabilitation [6, 41]. This may have important implications for the use of limb symmetry indices to assess function of the reconstructed limb [33], as changes in the uninjured limb may cause practitioners to either over or underestimate the capacity of the reconstructed limb. For example, by approximately 9‐months post‐surgery, single leg CMJ jump height in the ACLR limb reaches values similar to the baseline values of the uninjured limb (Figure 2, Table 1). However, a significant between limb asymmetry was present indicating that the uninjured limb also increased jump height over time (Figure 5). While these issues have been highlighted when assessing lower limb strength [6, 41], our findings show they also extend to measures of single leg jump performance. As such, practitioners should be mindful of the limitations of limb symmetry indices when used as a marker of rehabilitation status. Normative values for lower limb strength and vertical jump performance of age, sex, and sport matched populations may provide clinicians with additional targets for RTP criteria.

Out of all possible metrics, single leg CMJ jump height best distinguished between low and high ACL‐RSI scores. In our cohort, participants who achieved a jump height of less than 8.4 cm in the ACLR limb had a lower ACL‐RSI score (35 vs. 72) than those with greater jump heights at any time‐point across rehabilitation, with jump height explaining 67% of the variance in ACL‐RSI score. In contrast, previous work found little to no relationship between single leg CMJ jump height and ACL‐RSI [42] at the time of RTP from ACLR (~9‐months). Prior work has shown ACL‐RSI to be significantly associated with risk of secondary injury [38, 39]. A score of 76.7 (based on receiver operator curve) identified young athletes who sustained a recurrent rupture with 90% sensitivity [38]. Further, fear has been identified as a unique contributor to athletes not returning to preinjury levels of sport [36]. The decision tree results suggest non‐restoration of single leg jump height may underpin lower ACL‐RSI scores which may potentially lead to an inability to RTP and/or increased risk of reinjury.

A strong negative association was observed between isometric knee extension peak force asymmetry and KOOS scores (Figure 6). Participants who displayed > 38% between limb asymmetry had significantly lower KOOS scores (65 vs. 82) at any time‐point throughout rehabilitation. Similar to ACL‐RSI, previous work has only investigated the relationship between lower limb strength and KOOS at, or after, the time‐point of RTP [4, 13, 51, 55], showing limited ability to differentiate those with higher and lower scores. The results presented here suggest an individual's perception of their knee function could be influenced—consciously or unconsciously—by the strength of the quadriceps in their uninjured limb. Alternatively, lower KOOS scores may be indicative of increased knee joint symptoms and pain, which may be limiting knee extension strength.

However, the results and cut‐point values should be interpreted with caution. Both 8.4 cm SLCMJ jump height and 38% knee extension asymmetry are well below the baseline means from our cohort (Table 1 and Figure 6), and previously reported data at the time‐point of RTP [23, 34]. Current RTP criteria suggest significant improvements in both values are needed prior to resuming unrestricted sports activities [33]. Further, the relationship between PROMs and measures of lower limb strength and vertical jump metrics has only been investigated through statistical inference. More causal study designs are needed to either confirm or deny if changing objective measures of function leads to a change in PROMs.

Given that this was an observational study, participants' training during rehabilitation was not controlled. Additionally, the lack of an age‐ and sex‐matched control group of individuals who did not undergo rehabilitation following ACLR limits the ability to conclude whether improvements in vertical jump performance and strength were due to rehabilitation or time alone. Future work is needed to investigate the optimal rehabilitation strategies and exercise interventions to increase lower limb strength and jump performance following primary ACLR. Further, the current study only tracked participants up to the 12‐month time‐point. Future work should examine whether these trends persist or plateau beyond the first year of recovery. The population in this study comprised young, recreationally active individuals. As such, the values presented should be interpreted with caution when used as ‘normative’ data applied to other populations, such as adolescent and elite athletes. Although this study is the first to track a group of individuals from 3‐ to 12‐months post primary ACLR, it is a relatively small cohort. Prior work has suggested graft type may influence lower limb strength [28] and vertical jump performance [34, 46]. While we were underpowered to perform any subgroup analysis, the majority of participants in this study received a hamstring graft (90%). We performed a sensitivity analysis and re‐ran all models after removing *n* = 3 participants who did not receive a hamstring graft, and this did not change any findings from the mixed models. Future studies should explore if graft choice, along with other demographic (e.g., sex and age) and surgical characteristics (e.g., meniscus repair and lateral extra‐tenodesis) influence the recovery trajectories of both strength and vertical jump metrics through the time‐course of rehabilitation. Additionally, future work might consider exploring the relationship between objective functional measures and other PROMS, including the International Knee Documentation Committee and Lysholm scales.

## CONCLUSION

This study provides novel insight into the recovery of vertical jump metrics, lower limb strength, and PROMs from 3‐ to 12‐months following primary ACLR. The results show a faster rate of recovery across most jump and strength metrics during the first approximately 6‐months of rehabilitation, followed by a slower rate up to 12‐months post‐operatively. Significant improvements in PROMS were also observed, and single leg CMJ jump height height and knee extension peak force asymmetry were identified as key discriminators between higher and lower ACL‐RSI and KOOS scores, respectively. These findings provide valuable benchmarks that may help inform rehabilitation through identification of athletes diverging from common recovery trajectories.

## AUTHOR CONTRIBUTIONS

**Benjamin Dutaillis** and **Mathew N. Bourne**: Concept and design. **Benjamin Dutaillis**: Data collection. **Benjamin Dutaillis**: Data analysis. **Benjamin Dutaillis**, **Tyler Collings**, **Philip Bellinger**, **Ryan G. Timmins**, **Morgan D. Williams**, and **Mathew N. Bourne**: Data interpretation. **Benjamin Dutaillis**, **Tyler Collings**, **Philip Bellinger**, **Ryan G. Timmins**, **Morgan D. Williams**, and **Mathew N. Bourne**: manuscript preparation.

## CONFLICT OF INTEREST STATEMENT

The authors declare no conflicts of interest.

## ETHICS STATEMENT

This study was approved by Griffith University Human Research Ethics Committee (Ref No: 2022/253). All patients provided written informed consent.

## Data Availability

Data is available upon request.
